# Monograph of the First Permanent Molar

**Published:** 1887-11

**Authors:** E. Audrien

**Affiliations:** Paris


### Monograph of the First Permanent Molar.

BY DR. E. AUDRIEN, PARIS.

(Translated by G. P. Terry.)

A SYNOPSIS.

The object of this paper is to prove first, that, anatomically, physiologically, and pathologically speaking the first permanent molar must be considered as a tooth of transition.

Second, that the wisdom tooth, although it does not occupy the space left by the extraction of the first permanent molar, may yet in a practical point of view, and on account of the pathological relations which exist between these two teeth, be regarded as the tooth of replacement of the first permanent molar.

Lastly, there are very many cases where the first permanent molar can and must be extracted for the advantage and health of the rest of the teeth.

The physiological role of the first permanent molar can be summed up in three points:

1. To limit the part of the maxillary arch occupied by the milk teeth during the period of their replacement by the corresponding permanent teeth.
2. To maintain the articulation of the jaws at the desired height during this replacement.
3. To serve for mastication during the shedding of the milk teeth.

Let us recapitulate these three points. As the first permanent molar has most always achieved its eruption before the beginning of the replacement of the milk teeth, as this replacement does not usually begin but by the seventh year only to terminate by the twelfth or thirteenth year; that is to say at the time when the second permanent molar achieves its eruption, it follows that the first pernanent molar finds itself placed between the milk teeth and the space which the posterior molars will occupy as an obstacle destined to hinder on one hand the second bicuspid from encroaching upon the posterior part of the maxillary arch, and on the other the second permanent molar from encroaching upon the anterior part of this arch.

It obliges these teeth (second permanent molars) as soon as they accomplish their eruption to distend and enlarge the maxillary arch, which is one of the conditions of the good arrangement of the denture.

The anterior part of the maxillary arch must enlarge considerably, since all the replacement teeth, less the second bicuspid, are more voluminous than the corresponding milk teeth, and that the surplus of volume of the second temporary cannot be compared to the excess of size of the incisors and permanent canines in relation to the corresponding temporary teeth. We do not speak of the first bicuspid nor of the first temporary molar which have almost same diameter of a contiguous condition in respect to the other, the and which, consequently, does not change the space. As to the posterior part of the arch, it develops itself as soon as the two posterior permanent molars appear. It is in this way at least that things progress when the dentition is accomplished in a normal manner; when the teeth are of good quality and remain so.

It seems to us useless to insist upon the role which the first permanent molars play in regard to the maintaining of the height of the articulation. It is evident that, during the replacement of the temporary molars and before the first bicuspid, the second permanent molar has achieved its eruption, if the first permanent molar had not existed the height of the articulation would be uncertain. The antagonist teeth would have faulty occlusion, the one upon the other, according to the degree of advancement of the eruption of the bicuspids, and that in short, mastication would be very difficult; which happens, nevertheless, when one is obliged to extract at an unfortunate period the permanent first molars, from whatever motive.

Lastly, while the canines and the molars of the temporary set are shaky and about to fall, or rather when they have been shed before the appearance of the corresponding permanent teeth, or rather again before the first becuspids have attained their full height, they alone serve the child to chew the slightly hard foods until the second permanent molars and the second bicuspids have accomplished their eruption; from which we draw the conclusion, that in a physiological point of view *the first permanent*

*molar is of extreme usefulness from its eruption until the eruption of the neighboring teeth*, a time at which we believe to be able to prove that its presence in a great number of cases is less useful and even often injurious; that in one word, it constitutes an organ of transition between the deciduous and permanent teeth, and that in persuance of this, once its role accomplished, one need not fear its extraction, since the circumstances demand it.

### FREQUENCE OF THE DECAY OF THE FIRST PERMANENT MOLAR AND OF THE WISDOM TOOTH.

But, before approaching the question even of the fitness of this extraction, there is a point which governs it and upon which we must call attention; it is the extreme frequency of decay first in the first permanent molar and then in the wisdom tooth.

Here is what we have ascertained at the "Hospice des Enfants Assistes" of Paris, in seven years, from 1863 to 1870.

Upon 1,000 children from nine to twelve years of age, of both sexes, whose mouths we inspected during that lapse of time, that is to say, 4,000 first permanent molars, 2,957 were more or less decayed, which one can easily verify in looking over our statistics.

HOSPICE DES ENFANTS ASSISTES.

1,000 subjects (6 not recorded) from 9 to 12 years old.

| MOUTHS. | | | | |
|---|---|---|---|---|
| With 4, first per. molars decayed. | With 3, first per. molars decayed. | With 2, first per. molars decayed. | With 1, first per. molar decayed. | With no first per. molars decayed. |
| 410 | 298 | 155 | 113 | 24 |

By multiplying each number of mouths by the corresponding number of decayed teeth it is found that

| | | |
|---|---|---|
| 410 x 4 equals | 1640 | 2,957 or 74 per cent. |
| 298 x 3 " | 894 | |
| 155 x 2 " | 310 | |
| 113 x 1 " | 113 | |

But, could one object to this true record for these children foundlings, that is to say for children who are generally of a bad constitution, scrofulous, etc., and who have not been cared for at all, that this can not be certainly applicable to children brought up in comfort?

Well, it is an error, for if, in regard to these statistics, we bring forth that which we have made in our city practice from 1872 to 1880, that is to say, a city practice where the children, if it be allowable to express one's self in that way, are rather too well taken care of, than not well enough, and find them in well fed condition, we will see that the proportion of decayed first permanent molars with these children is yet a little larger than in those other children. Out of 600 mouths from nine to twelve years old, that is to say out of 2,400 first permanent molars, 1784 were decayed, being more than 74 per cent.

See the following table, city practice, 600 cases irrespective of sex, from nine to twelve years old.

| MOUTHS. | | | | |
|---|---|---|---|---|
| With 4, first per. molars decayed. | With 3, first per. molars decayed. | With 2, first per. molars decayed. | With 1, first per. molar decayed. | With no first per. molar decayed. |
| 281 | 128 | 101 | 74 | 16 |

Or, 281 x 4 equals 1,124

128 x 3 " 384

101 x 2 " 202

74 x 1 " 74

} 1,784 or 74.33 per cent.

There is the simple fact indicated by figures gathered with care. At least this is the result as we have observed it in Paris, the only place where we had occasion to make the researches.

And if now, instead of having to do with mouths of nine to twelve years old, we carry our investigations into mouths of persons over fifty years of age, that is to say, of persons in whom the eruption of the permanent teeth happened at a period when the only remedy for dental decay was, so to speak, the extraction, we find a more surprising result.

It is rare to find first permanent molars, where, if they are present they are either filled or decayed, which is easy to see by the following statistics.

City practice 100 cases (men and women) from 55 to 65 years old.

| MOUTHS. | | | | |
|---|---|---|---|---|
| With 4 first per. molars absent. | With 3 first per. molars absent. | With 2 first per. molars absent. | With 1 first per. molar absent. | With 4 first per. molars present. |
| 41 | 31 | 17 | 11 | 2 |
| The | remaining | 1st per. molar | are healthy | or filled. |

Upon 400 first permanent molars—

| | | |
|---|---|---|
| 41 x 4 equals 164 | | |
| 31 x 3 " 93 | | |
| 17 x 2 " 34 | | 302 extracted teeth, 75½ per cent. |
| 11 x 1 " 11 | | |

There is here an indisputable fact: The first permanent molar decays in the proportion of from 74 to 75 per cent. and it is with this fact that we think we must count on the good arrangement and the preserving of the other teeth. After the first permanent molar, it is the wisdom tooth which decays the most frequently and we will soon mention what relation exists between the presence or absence of the first permanent molar, and the decay or health of the wisdom tooth. The second permanent molars are next liable to decay, then the bicuspids, then the large and small incisors, and last, the canines.

But we have not to speak upon the prequency of the decaying of these last teeth, we must confine ourselves to what concerns the first permanent molar and after it the wisdom tooth.

We know perfectly well that the statistics which we have given do not exactly agree with those of some of our contemporaries with which we have been able to compare. It is true that the points to *prove* being not absolutely identical the relations were difficult enough to establish. Was it the difference in country and city practice? We cannot account for it, but what we can affirm, is that since twenty-five years that we occupy ourselves with all that pertains to the decay of the first permanent molar and wisdom teeth, all the statistics we have made concerning this subject have given almost identical results. We believe, therefore, that we can rely upon them.

### CAUSES OF THE FREQUENCE OF THE DECAY OF THE FIRST PERMANENT MOLAR.

But what are the causes of the frequent decay of the first permanent molar?

1st. It is not as dense as the rest of the permanent teeth.

2d. The external confirmation of its crown.

3d. The constant acidity of the fluids of the mouth during the replacement of the milk teeth.

4th. The neighborhood of the second temporary molar almost always deteriorated for some time before its shedding.

According to Dr. Galippe the density of the first permanent molar is greater than that of the milk teeth, but less considerable than that of the other permanent teeth, or, as according to that author, the resistance of the teeth to decay varies in one person with the different types of teeth, being more or less dense, it follows that the coefficient of resistance against decay of the first permanent molar is less than those of the other permanent teeth, which are more dense.

In regard to their exterior form, everybody knows that the grooves which are found upon the grinding surface of the first permanent molar between the cusps are so to speak without enamel or rather are the seat of deep fissures, whence the facility of the alteration of the tooth. And as the children clean their teeth but liltle and in certain classes of society eat candies, etc., it results that the food debris lodges in all the dental fissures which they meet, remain there, acidify, and produce the ravages inherent to the action of acids on the teeth.

But at the period of the replacement of the teeth there are not only the fissures or the natural cavities of the teeth which give rise to the acidity; there is at that time another cause more powerful to give rise to the acidity; it is the interstices which the shaky temporary teeth cause up to their shedding between them and the gums, insterstices where food debris lodges and ferments rapidly. Moreover, the decayed temporary teeth that one does not take the pains to fill, because one thinks in those cases the operation is useless, be it that one neglects it, become just as much the seat of infection, and in the whole mouth there follows a *general* acid reaction, very easy to determine by litmus paper. Concerning the deleterious action of the temporary molar upon the first permanent molar, I have never had a doubt. In almost every instance when a temporary molar is decayed on its posterior surface, there will be found decay on the anterior surface of the first permanent molar. Such a condition is an indication for its extraction.

(*To be Continued.*)

